# Supplementary material for: In vivo imaging of β-cell function reveals glucose-mediated heterogeneity of β-cell functional development
Source: eLife. 2019 Jan 29;8:e41540. doi: 10.7554/eLife.41540 (PMC6395064; doi:10.7554/eLife.41540)
Supplement: Supplementary file 1. — The table shows comparison of developmental process and critical stage in zebrafish and mouse pancreas development. [file elife-41540-supp1.docx]

Supplemental Table 1. Comparison of the critical events and the time windows in zebrafish and mouse pancreas development.

| **Comparison of events and critical time window in zebrafish and mouse pancreas development** | | | | | |
| --- | --- | --- | --- | --- | --- |
| Developmental  process | **Zebrafish** | |  | **Mouse** | |
|  | Events | Stage |  | Events | Stage |
| Pancreatic specification | *Pdx-1* appears in pancreatic primordial (Tiso et al) | 10 somite |  | *Pdx-1* appears in dorsal endoderm (Tiso et al) | E8-E8.5 |
| Pancreatic budding | emergence of *insulin* gene (Shih et al) | 15 somite |  | dorsal bud appears (Avolio et al) | E8.5-E9.5 |
|  |  |  |  | α-cells appear first (Yamaoka & Itakura) |  |
|  | dorsal bud appears (Shih et al) | 24hpf |  | ventral bud appears ((Avolio et al)  β-cells and δ–cells appear (Yamaoka & Itakura) | E9.5-E10.5 |
|  | emergence of *glucagon* gene (Shih et al) | 22-26hpf |  |  |  |
|  | ventral bud appears (Shih et al) | 32-40hpf |  |  |  |
| Rotation and  buds fusion | fusion of dorsal bud and ventral bud (Shih et al)  emergence of *ghrelin* and *trypsin* genes (Shih et al) | 48-52hpf |  | tip and trunk domain formation ((Avolio et al)  trunk domain has Nkx6.1, Sox9 ((Avolio et al)  scattered islet aggregates formation ((Avolio et al) | E12.5-E14.5 |
| Islet morphogenesis | complete fusion of the two buds (Shih et al)  principal islet forms core-mantle structure (Shih et al) | 52-72hpf |  | condense islets form core-mantle structure (Tiso et al) | E18.5-E20 |
| Islet vascularization | initiation with aorta contacting principal islet  perfusion with blood flow in most vessels  fully vascularized principal islet | 36-44hpf  48-60hpf  66-72hpf |  | initiation with endocrine cell differentiation (Reinert et al)  perfusion with blood flow in most vessels (Shah et al)  completed islet vascularization (Reinert et al) | E13.5  E14.5  E20-at birth |
| β-cell differentiation | emergence of insulin-positive cells (Shih et al)  β-cell proliferation (independent of Cn/NFAT)  appearance of glucose-responsive β-cells  Further enhancement of embryonic β-cell function | 15 somite  36-40hpf  44-48hpf  48-72hpf |  | emergence of insulin-positive cells (Yamaoka & Itakura)  β-cell expansion (independent of Cn/NFAT) (Shah et al)  appearance of glucose-responsive β-cells (Reinert et al)  β-cell functional maturation (Qiu et al) | E9.5-E10.5  E14.5  P2  P9-P15 |
